# Role of* Helicobacter pylori* Eradication Therapy on Platelet Recovery in Chronic Immune Thrombocytopenic Purpura

**DOI:** 10.1155/2017/9529752

**Published:** 2017-01-17

**Authors:** Khan Sheema, Ujjan Ikramdin, Naz Arshi, Naz Farah, Sheikh Imran

**Affiliations:** ^1^Liaquat University of Medical and Health Sciences, Jamshoro, Pakistan; ^2^National Institute of Blood Diseases and Bone Marrow Transplantation, Karachi, Pakistan

## Abstract

*Background*. Idiopathic thrombocytopenic purpura (ITP) is a bleeding disorder in which the immune system destroys native platelets. In this condition an autoantibody is generated against a platelet antigen. ITP affects women more often than men and is more common in children than adults.* Objective*. To assess the effect of* Helicobacter pylori* eradication therapy (HPET) on platelet count in* Helicobacter pylori* associated chronic immune thrombocytopenic purpura (chronic ITP) in adult.* Materials and Methods*. It is an interventional prospective study conducted at Liaquat University of Medical and Health Sciences, Jamshoro, from 2014 to 2015. A set of 85 patients diagnosed with chronic ITP were included in the study via convenient sampling. Patients with platelets count < 100 × 10^9^/L for >3 months were selected. They were posed to first-line investigations which comprised complete blood count (CBC) and peripheral blood smear examination followed by second-line tests including bone marrow examination and* Helicobacter pylori* stool specific antigen (HpSA-EIA). Standard* H. pylori* eradication therapy was offered and the patients were assessed at regular intervals for 6 months.* Results.* Of the 85 study patients, 32 (37.6%) were male and 53 (62.3%) were female. Mean ages of* H. pylori* positive and negative subjects were 43.89 ± 7.06 and 44.75 ± 7.91 years, respectively. Bone marrow examination confirmed the diagnosis and excluded other related BM disorders.* H. pylori* stool antigen (HpSA) was detected in 34 (40%) patients and hence regarded as* H. pylori* positive; the rest were negative. Treatment with eradication therapy significantly improved the mean platelet counts from 48.56 ± 21.7 × 10^9^/l to 94.2 ± 26.8 × 10^9^/l.* Conclusion.* We concluded that the* anti-H. pylori* eradication therapy improves blood platelet counts in chronic immune thrombocytopenia.

## 1. Introduction

Idiopathic thrombocytopenic purpura (ITP) is an autoimmune phenomenon, hence also called immune thrombocytopenic purpura (ITP). In this disease antibody coated platelets are phagocytosed by immune cells [1]. ITP may be categorized as recently diagnosed ITP, persistent ITP (3–12-month duration) and chronic ITP (≥12 months) [2, 3]. Association between* H. pylori* and ITP has already been established.* H. pylori* is also associated with similar autoimmune disorders like pernicious anemia, rheumatoid arthritis, and sicca syndrome [4].

Morphologically,* H. pylori* is a Gram-negative spirochete which colonizes the stomach mucosa.* H. pylori* infection is implicated in the pathogenesis of gastric and duodenal ulcers. Persistent or recurrent* H. pylori* infection is a known risk factor for gastric lymphoma and adenocarcinoma [5].* H. pylori* eradication therapy (HPET) has shown promising results in the management of peptic ulcers, chronic gastritis, and even regression of lymphoma of stomach [6]. Pathogenetic mechanisms involved in* H. pylori* infection include mucosa adhesion, flagella, and urease production. Cytotoxic associated gene A (CagA) and vacuolating cytotoxin A (VacA) are proven virulent factors which interact with gastric mucosa for colonization and cause infection by* H. pylori* [7]. Cell wall lipopolysaccharide and neutrophil attracting protein (HP-NAP) also participate in the virulence chain [8, 9]. Chronic immune thrombocytopenic purpura (chronic ITP) is linked as one of the extraintestinal manifestations of* H. pylori* disease [10]. Chronic ITP is reported to improve by effective* H. pylori* eradication therapy (HPET) [11, 12]. The role of HPET however remains controversial as some studies have reported an improvement in platelet count but others have failed to demonstrate any benefit [4, 13, 14]. This study was specifically designed to evaluate effect of* H. pylori* eradication therapy (HPET) on platelet count recovery in chronic ITP patients presenting at the Liaquat University of Medical and Health Sciences (LUMHS), Jamshoro, Sindh.

## 2. Materials and Methods

This interventional and prospective study was conducted in compliance with Helsinki's declaration 2000 and with an approval by institutional ethical committee at LUMHS, Jamshoro, from 2014 to 2015. A total of 85 subjects were recruited through convenient sampling. Written informed consent was taken. The inclusion criteria comprised diagnosed cases of chronic ITP. Patients with major comorbidities, concomitant malignancies, other autoimmune phenomenon, or aplastic anemia were excluded. A comprehensive questionnaire, including detailed history and physical examination, was filled out for each patient by a clinician.

Venous blood from each patient was collected into two EDTA containing sample collection tubes, each one 3 ml in volume. Complete blood count (CBC), including hemoglobin (Hb) estimation and platelet count, using automated cell analyzer (Sysmex XN 1000i Tokyo, Japan) and peripheral blood smear examination, stained with Leishman's stain, were performed on all the samples.

After the collection of stool samples in clean containers, Rapid Strip HpSA (Rapid Immunochromatography) was performed for the detection of* Helicobacter pylori* antigens in stool.

For bone marrow examination, aspiration was carried out under local anesthesia (2% xylocaine); smear was prepared and stained to look for the number and morphology of megakaryocytes.

### 2.1. Standard* H. pylori* Eradication Therapy

Amoxicillin 1 gram 2x daily, Clarithromycin 500 mg 2x daily, and a proton pump inhibitor 40 mg 2x daily were given for a duration of 2 weeks. The eradication was confirmed with HpSA stool test on the 7th posttreatment day [16].

### 2.2. Follow-Up Assessment

All the study patients were followed for a period of 6 months. These were tested for CBC on weekly basis in the first month. Thereafter, weekly monitoring was continued in those on risk of bleeding (platelet count < 10,000/*μ*L), while the others were checked on a two-week basis. The disease response was categorized into the following three groups:

(a) Complete response (CR): platelet count ≥150 × 10^9^ l^−1^
(b) Partial response (PR): platelet count 50–150 × 10^9^ l^−1^
(c) No response (NR): platelet count <50 × 10^9^ l^−1^ or an increase of <20 × 10^9^ l^−1^ after at least 6 months of follow-up [15].

### 2.3. Statistical Analysis

Data analysis was performed on SPSS version 22.0. Kolmogorov-Smirnov test was used for normality of data for parametric variable analysis. Continuous variables were analyzed by student's *t*-test and categorical variables by Chi-square test. *P* level of significance was taken at ≤0.05.

## 3. Results 

**Figure 1:**
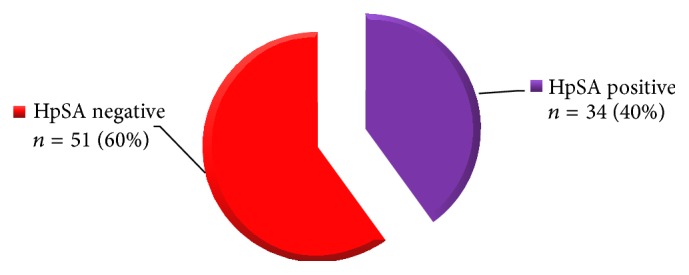
*H. pylori* infection in chronic immune thrombocytopenic purpura (*n* = 85).

A total of 85 patients were enrolled into the study; 32 (37.6%) were males and 53 (62.3%) were females.* H. pylori* positive cases were found to have a mean age of 43.89 ± 7.06 years, while the negative ones had a mean age of 44.75 ± 7.91 years. The parameters including Hb and hematocrit are documented in Table 1.* H. pylori* stool antigen (HpSA) was detected in 34 (40%) of subjects, and 51 (60%) were HpSA negative (Figure 1). Platelet counts before* H. pylori* eradication were noted as 48.56 ± 21.7 million/*μ*L which increased to 94.2 ± 26.8 million/*μ*L after eradication therapy (Table 2). All the study patients were followed up for a period of 6 months. A significant sustained response was found in* H. pylori* eradicated cITP cases. On the other hand,* H. pylori* negative cases showed only a mild transient response. Complete recovery (CR), partial recovery (PR), and no response (NR) of platelet recovery in HpSA positive cases (*n* = 34) were observed in 19 (55.8%), 10 (29.4%), and 5 (14.7%) cases, respectively (Table 3).

Bone marrow examination mostly showed increased number of megakaryocytes with abnormal morphology and distribution. Majority of the megakaryocytes were smaller in size. Other cell lineages showed normal morphology.

## 4. Discussion

An informal connection between* H. pylori *infection and immune thrombocytopenic purpura (ITP) is suggested by various clinical studies demonstrating platelet count response in approximately 50% of patients following* H. pylori *eradication [17]. Effects of* H. pylori *eradication therapy (HPET) on platelet recovery in chronic immune thrombocytopenic purpura were first reported by Gasbarrini et al. in 1998 [18]. An analysis of 25 reported series worldwide showed that eradication was successful in 671 of 792 (84.7%) patients [19]. Inaba et al. had reported similar findings as* H. pylori* eradication therapy improved platelet counts [20]. The authors of present study hold the view that* H. pylori *is linked with abnormal immune reaction producing platelet destruction. On the contrary, Stasi et al. and Gan et al. reported no improvement in circulating platelets after HPET [10, 21]. Geographical variations, sampling techniques and sample size, faulty study designs,* H. pylori strains*, drug compliance, drug efficacy, systemic bias, and research bias may be the main confounding factors.

The prevalence of* H. pylori* infection in chronic ITP patients varies markedly. We found* H. pylori *infection in 40% of the study cITP patients. A previous study, conducted in Italy, showed a* H. pylori* prevalence rate of 50% in cITP patients. In another study from Japan 75% of the cITP patients were found to have* H. pylori* infection. However, studies conducted in French and North American Caucasian cITP patients showed a low prevalence rate [19]. A prevalence rate of 50–80% has been marked in studies from Japan, Iran, and Korea [22–24]. It has already been shown that after HPET treatment the cITP of shorter duration responds better in comparison to the long standing ones [1, 21]. In the current study, complete response (CR), partial response (PR), and no response (NR) of platelet recovery in HpSA positive cases (*n* = 34) were noted in 19 (55.8%), 10 (29.4%), and 5 (14.7%) cases, respectively. The response found was statistically significant (*P* = 0.0001). Our findings of platelet recovery are consistent with those of previous studies [1, 15, 21]. All the study patients were followed up for a period of 6 months. A significant sustained response was found in* H. pylori* eradicated cITP cases. On the other hand,* H. pylori* negative cases showed only a mild transient response. Patients with PR and NR are speculated to have other aetiologies including HIV or HCV infection or have previously been treated with interferons or other antiviral therapy interfering with megakaryopoiesis.

Brito et al. [25] and Payandeh et al. [26] reported a recovery in platelet counts after HPET treatment at 6–12-month follow-up which is consistent with the current study.

## 5. Conclusion

We conclude that 40%* of the local cITP patients are H. pylori infected and that H. pylori* eradication therapy in these individuals significantly improves the platelet counts. Further studies on a larger cohort of patients, with a longer follow-up, will allow a better insight into the true prevalence of* H. pylori* infection and the duration of remission. Such studies will also conceivably endorse clarity of actual prevalence and understanding of mechanisms underlying the response to eradication therapy.

## Figures and Tables

**Table 1:** Baseline demographic and clinical characteristics of study patients (*N* = 85).

Parameter	HpSA positive	HpSA negative	*P*value^**∗**^
Age (years)	43.89 ± 7.06	44.75 ± 7.91	0.53
Male	12	20	—
Female	22	31	—
Hemoglobin (g/dl)	11.54 ± 1.68	12.19 ± 2.06	0.12
Hematocrit (%)	45.0 ± 7.5	47.0 ± 8.0	0.30
RBC (million/*µ*l)	2.91 ± 0.49	3.34 ± 0.55	0.0001
WBC (per *µ*l)	6283.7 ± 0.310	6239.0 ± 0.311	0.01
Platelets (× 10^3^/*µ*l)	12.3 ± 3.7	13.5 ± 4.1	0.9

HpSA: *Helicobacter pylori* stool antigen; *N*: total number of patients.

^**∗**^Based on Student's *t*-test.

**Table 2:** Platelet counts before and after *H. pylori* eradication therapy (*N* = 34).

Platelet counts	Mean	SD	*P* value
Before *H. pylori* eradication	48.56	21.75	0.0001
After *H. pylori* eradication	94.29	26.85	

*N*: number of patients.

**Table 3:** Evaluation of platelet counts in response to HPET in HpSA positive patients (*N* = 34).

Platelet response	Number	Percentile (%)	*P* value
Complete response (Cr)^*∗*^	19	55.8	0.0001
Partial response (Pr)^*∗∗*^	10	29.4	
No response (Nr)^*∗∗∗*^	5	14.7	

HPET: *H. pylori* eradication therapy; HpSA: *H. pylori* stool antigen.

^*∗*^Platelet count 150 × 10^9^ L^−1^.

^*∗∗*^Platelet count 50–150 × 10^9^ L^−1^.

^*∗∗∗*^Platelet count < 50 × 10^9^ L^−1^ or an increase of <20 × 10^9^ L^−1^ after at least 6 months of follow-up [15].
